# Supplementary material for: In Vivo Efficacy and Toxicity of an Antimicrobial Peptide in a Model of Endotoxin-Induced Pulmonary Inflammation
Source: Int J Mol Sci. 2023 Apr 27;24(9):7967. doi: 10.3390/ijms24097967 (PMC10178222; doi:10.3390/ijms24097967)
Supplement: Supplementary file 1 [file ijms-24-07967-s001.zip › ijms-2331670-supplementary.pdf]

SUPPLEMENTARY MATERIAL

S1. INHALATION EXPOSURE SYSTEM

The inhalation exposure system for mice used in the toxicity study is owned by Covance CRS LLC (now Labcorp Drug Development), Huntingdon, Cambridgeshire UK, the CRO where the experiments were performed**.** The system provided the determinations of achieved concentration of the peptide during the study and the particle size distribution. This is schematically represented in Figure S1. Briefly, the components of the system are:

- Exposure system: a flow through snout only chamber, an aluminium alloy construction comprising a base unit, one animal exposure section, a top section and a pre chamber
- Animal Restraint: a polycarbonate snout-only restraint tube
- Aerosol Generation: A single Aeroneb (Aerogen) vibrating mesh nebuliser was used per exposure system and a disposable clinical syringe driven by a syringe pump
- Inlet Airflow: from in-house compressed air system – breathing quality. Flow rate of 16 L/minute
- Extract Airflow: drawn by in-house vacuum system, filtered locally. Flow rate: 17 L/minute
- Airflow Monitoring: high quality tapered tube flowmeters, calibrated daily. In-line flowmeters monitored continuously

Mice were exposed to an atmosphere containing SET-M33. Different doses were achieved by varying the concentration of the peptide in the supplied formulation, the rate of formulation delivery to the aerosol generator and the system airflows. Animals were dosed once daily for 7 days. The administration takes 60 minutes each day. The animals under study were acclimatised to the method of restraint for 5 consecutive days immediately preceding their first exposure.

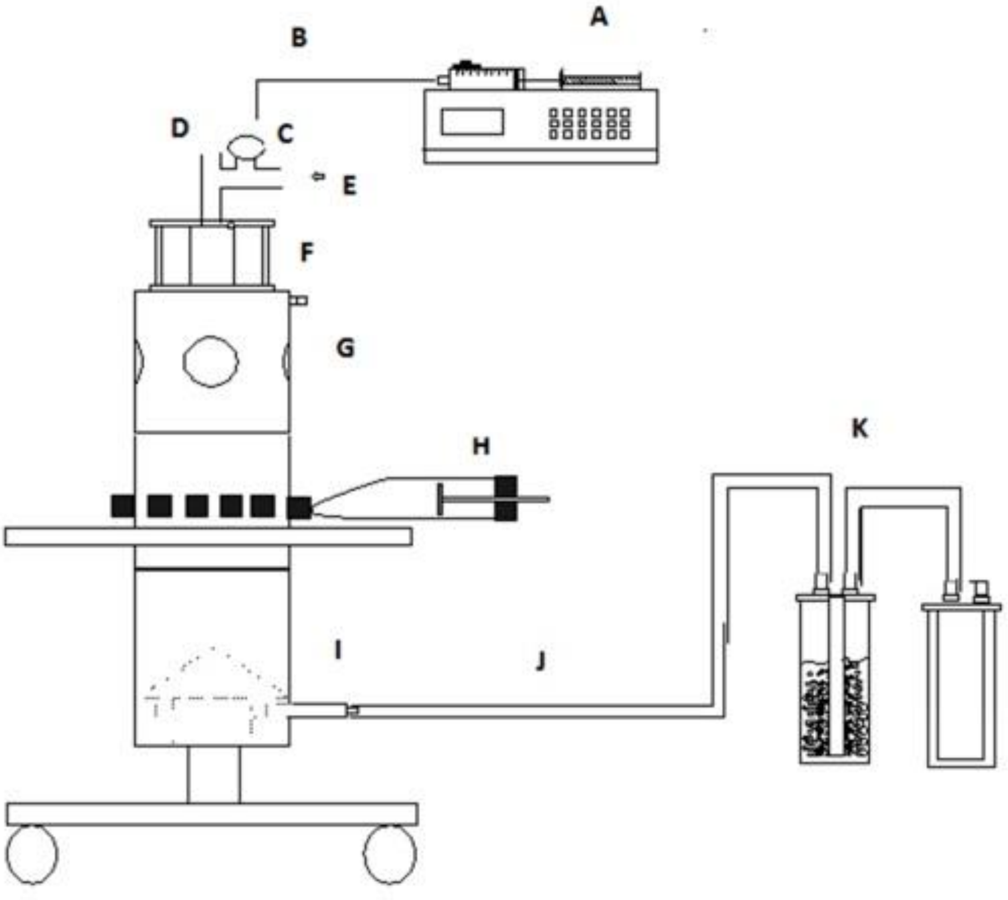

Key

A.	Syringe driver	G.	Inhalation exposure chamber
B.	Feed line	H.	Rodent restraint tube
C.	Nebuliser	I.	Chamber extract
D.	Balance air inlet	J.	Extract tubing
E.	Compressed air inlet	K.	Filtration units
F.	Aerosol conditioning pre-chamber		

Figure S1. Schematic representation of inhalation exposure system.

S2. UPLC conditions

Analytical column: Waters UPLC Peptide BEH C18, 130 Ǻ, 1.7 μm, 150 × 2.1 mm

Column temperature: 50°C

Mobile phase A: Acetonitrile/water/trifluoroacetic acid , 10/90/0.1 v/v/v

Mobile phase B: Acetonitrile/water/trifluoroacetic acid , 90/10/0.1 v/v/v

Linear gradient:

Time (min)	%A	%B
0.0	95	5
1.0	95	5
4.0	65	35
4.1	65	35
4.2	95	5
6.0	95	5

Flow rate: 0.25 mL/min

Detector: UV, 210 nm

Injection volume: 5 μL

Retention time: 3.8 min

The UPLC system was calibrated using external standards. Peak area data acquired by the data capture software using a 2nd order (quadratic) fit was subjected to least squares regression analysis.
